# Local treatment of colostomy prolapse with the MESH STRIP technique: A novel and highly efficient day hospital technique

**DOI:** 10.6061/clinics/2020/e1353

**Published:** 2020-01-06

**Authors:** Carlos Walter Sobrado Junior, Vivian Regina Guzela, Lucas Faraco Sobrado, Sérgio Carlos Nahas, Ivan Cecconello

**Affiliations:** Departamento de Gastroenterologia, Hospital das Clinicas HCFMUSP, Faculdade de Medicina, Universidade de Sao Paulo, Sao Paulo, SP, BR

**Keywords:** Stoma, Prolapse, Surgical Repair, Outpatient

## Abstract

**OBJECTIVE:**

Stoma prolapse is an intussusception of the bowel through a mature stoma. It can be caused by increased intra-abdominal pressure, excessively mobile bowel mesentery and/or a large opening in the abdominal wall at the time of stoma formation. It occurs predominantly in loop stomas, and correction methods include conservative modalities, such as local reduction to the prolapsed bowel, or surgical treatment. The purpose of this study was to describe our experience with the treatment of colostomy prolapse using a novel mesh strip technique.

**METHODS:**

Between February 2009 and March 2018, ten consecutive male patients underwent correction of colostomy prolapse under local anesthesia by peristomal placement of a polypropylene mesh strip. Operation time, short- and long-term complications, and recurrence rates were recorded and analyzed.

**RESULTS:**

No postoperative complications, morbidity or mortality were observed. The median length of the prolapse ranged from 6-20 cm, and the median operative time was 30 minutes. The median duration of follow-up was 25 months (range, 12-89 months). No relapse, mesh strip extrusion, local infection or granuloma formation were found.

**CONCLUSION:**

A simple, fast, and low-cost operation under local anesthesia using a mesh strip is a valuable option to treat colostomy prolapse.

## INTRODUCTION

**Figure 1:**
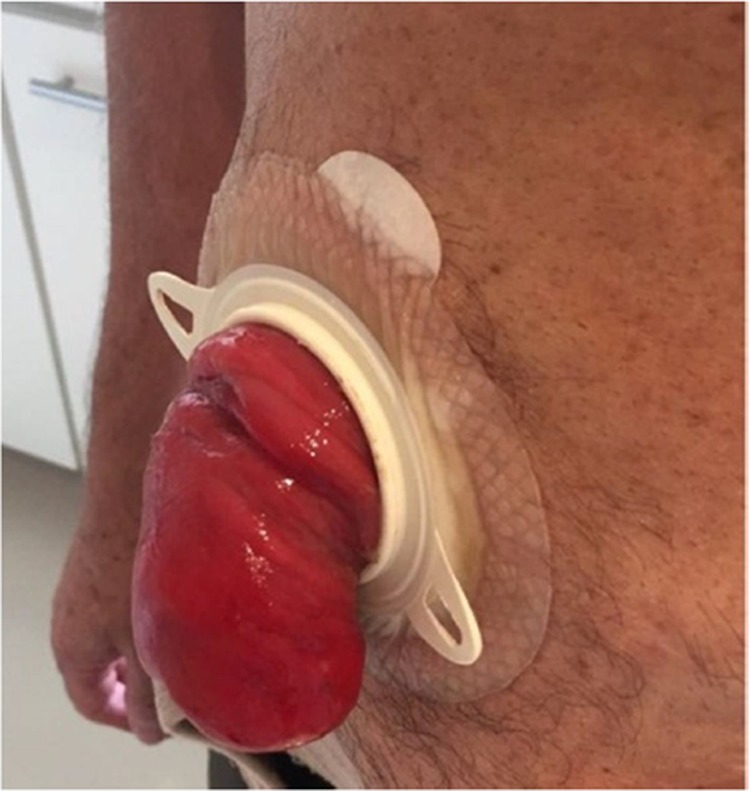
Prolapse before surgery.

Stoma prolapse is defined as intestinal intussusception through the stomal orifice (Figure 1). Its prevalence varies according to type, from 8% in end colostomies to 47% in loop colostomies, with the most common prolapse occurring in the distal limb (1). Ileostomies tend to prolapse at lower rates, with an incidence of approximately 2% (2).

The etiology of stoma prolapse is multifactorial, including increased intra-abdominal pressure caused, for example, by obesity, ascites or intracavitary expansive lesions. Excessively mobile mesentery can also contribute to prolapse formation, as can inadequate application of the colostomy/ileostomy surgical technique, with exaggerated opening of the wall for exteriorization of the loop.

The clinical picture of prolapse can range from aesthetic impairment and difficulty managing coupled devices, such as pouches and plugs, to bowel incarceration and strangulation.

Treatment is either conservative, with manual and postural reduction using lubricants or agents that reduce edema, or surgical. Among the surgical modalities, the existing approaches use abdominal or peristomal access, which may include resection of the prolapsed segment with a new anastomosis (manual or stapled), stoma reduction and reconfiguration or only reduction with fixation (1,3,4).

Outpatient surgeries are increasingly common because they contribute to a faster recovery and reduce the risk of in-hospital stays, such as nosocomial infection; moreover, the associated costs are lower (5,6). In Brazil, more commonly in the public health system, the high demand for treatment and the inversely proportional resource availability make outpatient surgery particularly attractive, as it expands the number of patients who can be treated without a large increase in the amount of necessary resources such as hospital beds.

Thus, to increase the treatment availability for patients with stoma prolapse, we describe a new outpatient surgical technique to correct this pathology using a polypropylene mesh strip.

## METHODS

This study was approved by the Ethics Committee of Hospital das Clinicas, Medical School, University of Sao Paulo. Written informed consent was provided by all patients of their own free will before the operation.

Ten consecutive male patients with stoma prolapse were selected and underwent the surgical treatment described between February 2009 and March 2018. Inclusion criteria were adult patients with a definitive prolapsed stoma and who were in good clinical health. Exclusion criteria were an ASA greater than 2, major abdominal wall defects that could interfere with the technique, current infection of the peristomal skin at the time of surgery or a prolapse greater than 20 cm. Bowel preparation is not necessary, and an 8-hour fast was the only requisite preoperative care. For the surgical technique, the patient is placed in horizontal dorsal decubitus position, and antibiotic prophylaxis is administered (nitroimidazole derivative and fluoroquinolone) as well as local asepsis and antisepsis with the positioning of the surgical drapes. After manual reduction of the prolapse, local anesthesia is administered; two punctures are made in the peristomal skin to infiltrate the anesthetic solution (2% lidocaine without vasoconstrictor) in a fan-like manner.

**Figure 2:**
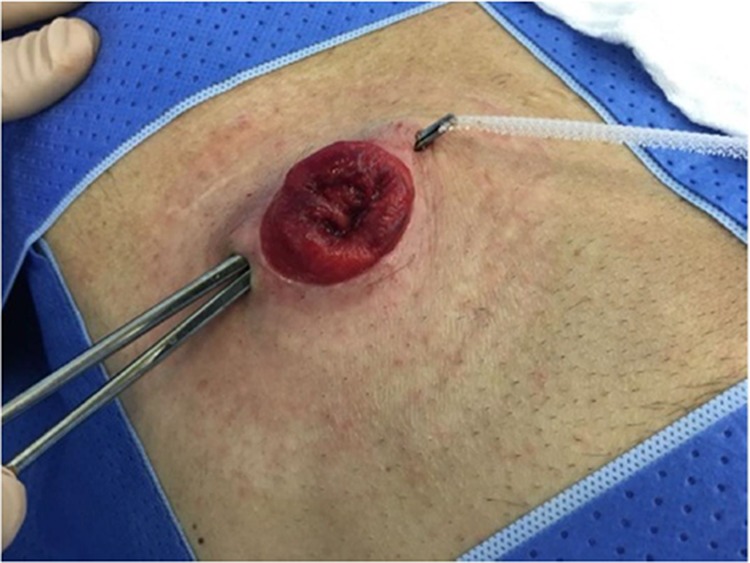
Passing the mesh around the stoma.

**Figure 3:**
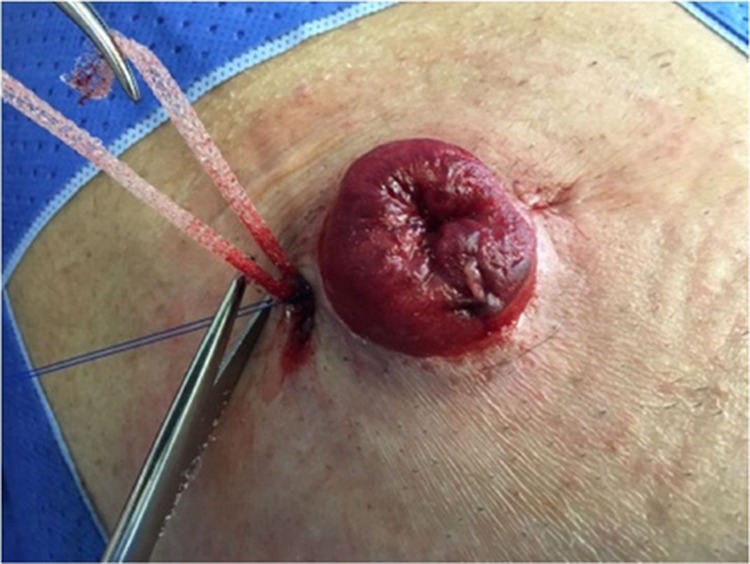
Adjustment of the peristomal diameter.

After local anesthesia, two diametrically opposite incisions of approximately 10 mm are made in the skin, approximately 3 mm apart from the stoma. The subcutaneous space is then dissected around the prolapsed stoma, making a tunnel circumferentially in this plane (Figure 2). A polypropylene mesh cut into a rectangular strip 1 cm in width × 10 cm in length is then placed inside the tunnel, circling the prolapsed stoma (Figure 3).

**Figure 4:**
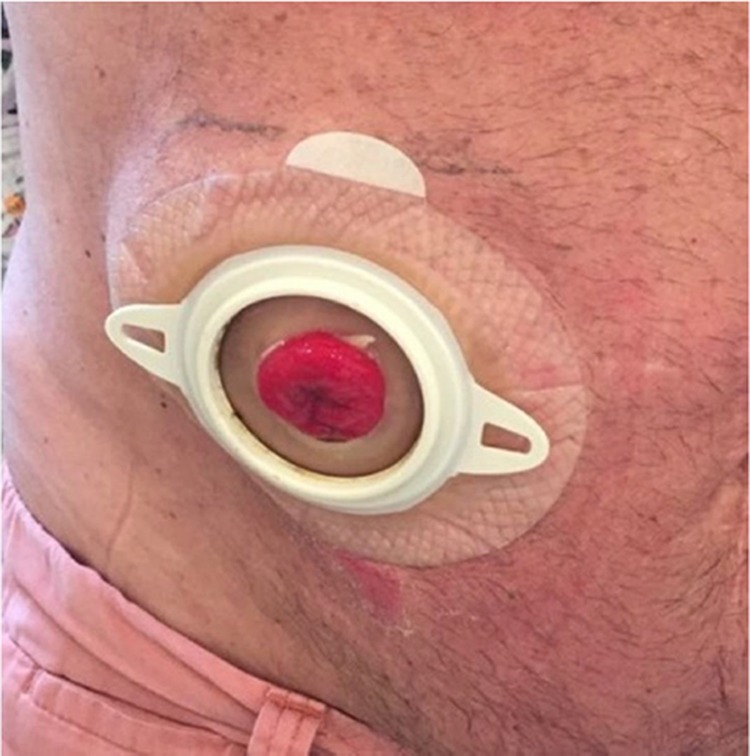
Twenty-eight days after surgery.

The ends are joined together, adjusting the diameter of the stoma so that both the vascular supply and patency are not compromised but keeping it sufficiently tight (one-finger diameter) so that prolapse does not recur. Next, the ends of the mesh strip are joined using simple non-absorbable sutures (Figures 3 and 4). The excess mesh strip is cut off, and the skin is closed.

**Figure 5:**
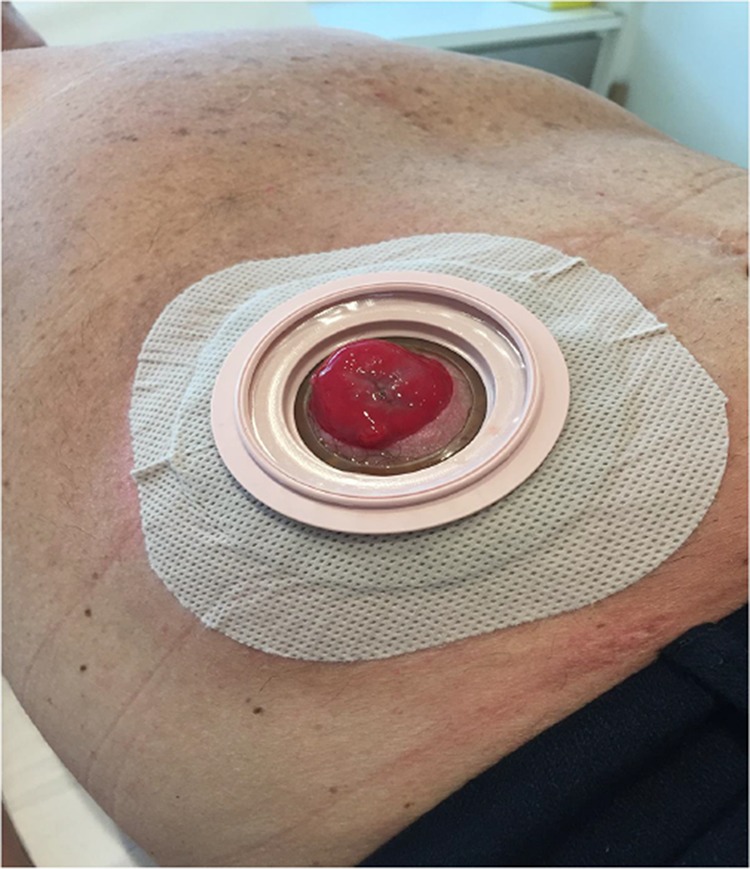
Twelve months after surgery.

The colostomy bag is adequately attached, allowing no stool extravasation at the operative site (Figure 5). Antibiotic prophylaxis is maintained for 24 hours by intravenous administration perioperatively and oral administration after hospital discharge.

## RESULTS

From February 2009 to March 2018, ten consecutive patients with colostomy prolapse underwent the mesh strip technique. The mean age was 51 years, with ages ranging between 37 and 78 years (Table 1). Only one of the participants had an end colostomy; the other nine had loop colostomies. All patients who underwent this technique had a maximum length of hospital stay of 8 hours. The median operative time was 30 minutes (range, 24 to 51 minutes), and the length of the prolapse ranged from 6 cm to 20 cm.

No immediate postoperative complications were noted, and all patients were discharged on the same day of surgery. Nine patients complained of mild local discomfort, which ceased after the administration of dipyrone (1 gram) or acetaminophen (750 milligrams) for 1-2 days. One patient had no complaints and did not use postoperative analgesics. Upon discharge, the patients were re-evaluated at 1, 3 and 12 weeks, as well as 12 months postoperatively or as requested. The median follow-up was 25 months (range, 12-89 months). No relapse, mesh strip extrusion or granuloma formation have been reported. No early or delayed postoperative interventions were required.

## DISCUSSION

Stoma prolapse is a common late complication. The incidence rate is variable and depends on systematic and long-term follow-up. Many intrinsic circumstances predispose patients to the development of prolapse; the most important factor in the prevention of colostomy prolapse is adherence to the basic surgical principles of stoma construction. Prolapsed and symptomatic colostomies require surgical treatment that can be performed by abdominal (laparotomy or laparoscopy) or peristomal access and may include resection with a new anastomosis (manual or stapled). Peristomal access with external segment resection and anastomosis with surgical stapling devices (linear or circular) have been widely used but have high costs, considering the use of technological devices (7). The technique described herein is low-cost and requires only a polypropylene mesh strip, costing an average of 15 times less than a stapler in Brazil. The short time to perform surgery and reduced hospital stay also contributed to decreasing the total cost of the treatment. The polypropylene mesh insert is important for reducing the risk of recurrence because it stimulates fibroblasts, leading to the formation of strong scar tissue. In this study, only patients with colostomies underwent surgery; however, patients with prolapsed ileostomies may also be eligible.

The scarcity of resources to fund the Brazilian public health system results in a decreasing number of surgical beds and a large number of patients with prolapsed stomas with no prospect of correction. In this setting, which offers a less invasive, low-cost surgical technique that does not require staples and that can be performed under local anesthesia in an outpatient unit (with expected discharge on the day of the procedure) can expand the reach of treatment. The new surgical technique, successfully applied in 10 patients, met the objectives of a short hospital stay, satisfactory postoperative recovery and absence of recurrence or complications. The procedure was performed safely and effectively in an outpatient unit under local anesthesia, allowing patients to return home early, providing a faster recovery and good acceptance of the method by all patients.

## CONCLUSION

A simple, fast, and low-cost operation under local anesthesia using a mesh strip is a valuable option to treat colostomy prolapse, avoiding the use of highly technological devices, and is achievable in day hospitals.

## AUTHOR CONTRIBUTIONS

Sobrado Junior CW was responsible for the study design, data retrieval and manuscript drafting. Guzela VR was responsible for the data retrieval and manuscript drafting. Sobrado LF collected and interpreted the data, as well as, generated the table. Nahas SC and Cecconello I were responsible for the critical analysis and manuscript review.

## Figures and Tables

**Table 1:** The baseline characteristics of the patients.

Sex	Male (10)
Age (years)	51 (37-78)
Body mass index	21.4 (19.8-31.7)
Systemic arterial hypertension	3
Diabetes mellitus	1
Symptoms	
Bulging	10
Pain	3
Bleeding	4
